# Prevalence of periodontitis and oral hygiene practices among diabetic and non-diabetic patients in a tertiary hospital in Lagos: a cross-sectional study

**DOI:** 10.11604/pamj.2023.45.131.37904

**Published:** 2023-07-19

**Authors:** Kehinde Olubukola Ojo, Oluwakemi Ololade Odukoya, Patricia Omowunmi Ayanbadejo, Damilola Akinlawon

**Affiliations:** 1 Department of Dentistry, College of Medicine and Health Sciences, Afe Babalola University, Ado-Ekiti, Ekiti State, Nigeria,; 2 Department of Community Health and Primary Care, College of Medicine, University of Lagos, Lagos, Nigeria,; 3 Department of Preventive Dentistry, Faculty of Dental Sciences, College of Medicine, University of Lagos, Lagos, Nigeria,; 4 University of Lagos Medical Center, University of Lagos, Lagos, Nigeria

**Keywords:** Periodontitis, non-diabetic patients, diabetic patients, oral hygiene, oral hygiene practices

## Abstract

**Introduction:**

periodontitis is the sixth leading long-term complication of diabetes mellitus which can impair diabetic patients' metabolic control. Patients with both diabetes mellitus and periodontal disease present with the challenge of managing these two chronic diseases, each of which may impact the other. The aim of this study was to determine and compare the prevalence of periodontitis and oral hygiene practices among diabetic and non-diabetic patients attending a large tertiary hospital in Lagos, Nigeria.

**Methods:**

this was a cross-sectional comparative study involving 110 diabetics and 110 non-diabetic patients aged 40 years and above. They were recruited from the diabetes and general medical out-patient clinics respectively in a large tertiary hospital in Lagos using a systematic sampling method. Data was collected using an interviewer-administered questionnaire. In addition, blood tests for glycated haemoglobin and oral examination using a simplified periodontal examination were conducted. The prevalence and severity of periodontitis and oral hygiene practices were compared between both groups. Data were analyzed with IBM SPSS version 21 Software.

**Results:**

the prevalence of periodontitis was higher among the diabetics 100 (90.9%) compared to the non-diabetic patients 79 (71.8%), and this was statistically significant (p<0.001). Severity of periodontitis among both groups was also statistically high 54 (49.1%) vs. 35 (31.8%) p<0.001.

**Conclusion:**

the prevalence of periodontitis was higher and more severe among diabetics compared to non-diabetics. Oral hygiene practices in both groups are not statistically significant p>0.05. Oral health education programs targeted at diabetic patients should be carried out to prevent and control periodontitis.

## Introduction

Periodontitis is a chronic inflammatory disease resulting in the destruction of connective tissues, alveolar bone, gingival bleeding, compromised aesthetics, recurrent periodontal infections, tooth mobility, unsatisfactorily digested foods, and eventual tooth loss. These may all have negative impacts on quality of life, with implications for function, comfort, self-confidence, social interactions, and food choices [1]. Diabetes mellitus (DM) is a group of metabolic diseases characterized by hyperglycemia due to a total or relative lack of insulin secretion and insulin resistance or both according to the World Health Organization (WHO) [2]. The abnormalities involved include carbohydrate, protein, and fat metabolism. Both diabetes and periodontitis are chronic diseases. Diabetes results in changes in the function of immune cells and impaired defects in this first line of defense against periodontal pathogens which can facilitate bacterial persistence in the periodontal pocket and significantly increase periodontal inflammatory-induced destruction [3].

**Figure 1:**
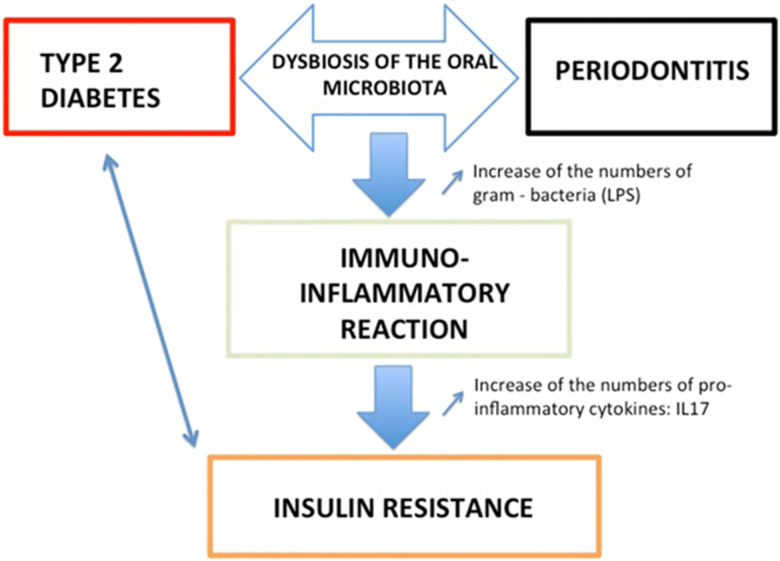
bidirectional link and explanation between type 2 diabetes and periodontal diseases

There is emerging evidence to support the existence of a two-way relationship between diabetes and periodontitis, with diabetes increasing the risk for periodontitis, and periodontal inflammation negatively affecting glycaemic control [4]. This indicates the reciprocal relationship that exists between diabetes and periodontitis with evidence to suggest that periodontal disease may increase the risk of poor metabolic control [5] or that periodontal disease has the potential to adversely impact glycaemic control in patients with diabetes mellitus [6]. Type 2 diabetes is associated with periodontitis and will induce an immune systemic reaction that could trigger insulin resistance (Figure 1).

In Nigeria, several studies have been conducted to assess the prevalence of periodontitis among patients with diabetes, however, most of them are still of a cross-sectional descriptive nature, making it difficult to identify the true prevalence among diabetic patients compared to those without diabetes [6-9]. This study was therefore carried out to assess and compare periodontitis and its severity among diabetic and non-diabetic adults. This will provide data to establish an updated estimate of those diabetics affected by periodontitis and to see if there has been an improvement since previous similar studies have been done in the same location [10-12]. It may help in the prevention and control of both periodontal disease and diabetes mellitus.

## Methods

**Study area and design:** the study was carried out at the diabetes clinic and general outpatient clinic of a large tertiary hospital in Lagos. The diabetes clinic provides consultation, treatment, and follow-up for patients with high blood glucose levels. The general outpatient clinic (Mondays, Tuesdays, Thursdays, and Fridays) is the first contact for consultation of all patients who present for routine checkups and other complaints. This was a cross-sectional comparative study among two study groups; type 2 adult diabetes patients attending the diabetes clinic and non-diabetic patients who presented at the general outpatient clinic for other reasons excluding diseases associated with diabetes mellitus. The inclusion criteria for diabetic patients were; known type 2 diabetes patients diagnosed by the health care practitioner for more than one year [13], patients aged 40 years and above [13], and patients with at least 3 teeth in each quadrant. For the non-diabetic patients, patients with normal blood glucose levels <100mg/dl and without any clinical symptoms (polyphagia, polyuria, polydipsia, paraesthesia, poor healing, pruritus, numbness, blurry vision, weight loss) suggestive of diabetes who were at least 40 years of age were included.

**Sample size:** the minimum sample size for each group was determined using the formula for comparison of proportions [14]. The proportions of periodontitis in diabetic and non-diabetic patients were 43.7% [15] and 25% [15] respectively from previous studies. Using a confidence interval of 95% and a power of 80%, it was found that the minimum sample size for each group was 97. This was increased by 10% to make allowances for non-responses and incomplete questionnaires. The final minimum calculated sample size for each group was 110.

**Sampling technique:** subjects were recruited using a systematic sampling method (using the diabetes and general outpatient clinic lists for the diabetic and non-diabetic participants respectively) by calculating a kth interval and selecting eligible consenting patients till the sample size was reached.

**Data collection:** data were collected using an interviewer-administered questionnaire which was however divided into sections A-E. Section A assessed socio-demographic and socioeconomic characteristics of all participants, section B assessed the oral hygiene habits, section C assessed participants´ blood sugar profile, section D assessed the knowledge of the periodontal disease, while section E assessed the periodontal status of all participants for the period of 6 months (between February and August 2018).

**Clinical examination:** one of the side rooms was used for the interview and oral examination to afford the patients some privacy in order to prevent other patients from listening to the interview which could influence their responses. Oral examination was carried out on visual inspection and palpation, adequate light source and strict adherence to universal precautions using sterile mouth mirror and probes, and instruments for assessing periodontal disease status such as (CPITN-E) probes using the Community Periodontal Index of Treatment Needs (CPITN). This is primarily a screening examination that includes clinical assessment for the presence or absence of true periodontal pockets (destruction of tooth-supporting tissues), calculus, and gingival bleeding. This index uses a special periodontal probe (a specially designed lightweight probe with a 0.5mm ball tip bearing a black band between 3.5-5.5 mm from the ball tip) for epidemiological purposes in adult populations and 10 specified index teeth (17, 16, 11, 26, 27, 36, 37, 31, 46, 47) were examined [16]. The patient´s mouth was divided into sextants: 4-7 (upper right); 3-3 (upper front); 4-7 (upper left); 4-7 (lower left); 3-3 (lower front); 4-7 (lower right). The scores of the CPITN were considered as follows [17].

**Scores and criteria:** 0: no periodontal diseases, healthy gum. 1: bleeding observed during probing. 2: calculus with gingival bleeding. 3: the presence of pathological pocket, 4-5mm. A gingival margin was found on the black area of the probe. 4: pathological pocket 6mm deeper, black area of probe no longer visible. 5: excluded sextant (less than two teeth present). The prevalence of periodontal disease among diabetic patients was categorized according to their periodontal status whereby those with the highest scores of CPITN 3 or 4 were in the severe periodontal disease group (periodontitis), while those with CPITN 1 or 2 were in the mild periodontal disease group (gingivitis) [18]. Those with CPITN 0 were said to have healthy gingiva. The procedure for each patient took about 10 minutes. All participants were tested for fasting blood sugar using a glucometer. All completed questionnaires were collected and appropriately coded immediately after. All patients who presented with periodontal diseases were referred to the periodontology clinic for management and follow-up.

**Oral hygiene practice:** it is the exercise of keeping one's mouth clean and disease free. It involves brushing twice daily, flossing once daily, healthy eating, and regular visit to the dentist as well as six monthly scalings and/or polishing [19]. Optimal oral hygiene practices are instrumental to good dental, lingual, and gingival health generally. However, in some situations, it may be challenged by ignorance, poverty, erroneous beliefs, and cultural practices [20]. According to the Centre for Disease Control (CDC), optimum oral hygiene practice is brushing twice daily, flossing daily between teeth, and visiting the dentist at least once annually [21]. In Nigeria, the toothbrush is sometimes replaced by a chewing stick or used in combination among some individuals. Assessment of oral hygiene practice in the study included type of oral hygiene aid, frequency of cleaning, method of cleaning, whether flossing is done, visitation to the dentist, and whether scaling is done regularly.

**Blood sample collection for glycated haemoglobin:** two milliliters of venous blood samples were drawn from the cubital vein of all diabetic patients under aseptic conditions by the investigator and sent to the chemical pathology laboratory to determine the glycated haemoglobin level. According to the American Diabetes Association standard of care for diabetic control, patients with HbAIc less than 7% were considered good (glycaemic control), while patients with equal to and greater than 7% were considered poor [22].

**Data entry and analysis:** this was done using the IBM SPSS version 21 Software. Chi-squared and Fisher´s exact tests were used to test the relationship between the outcome and independent variables. 1) The prevalence of periodontitis amongst diabetics was acquired by calculating the number of diabetic patients in this study who had the periodontal disease at the time of study and the percentage of that number out of the total number of diabetics involved in the study. The prevalence of periodontitis amongst non-diabetics was acquired by calculating the number of non-diabetic patients in this study who had the periodontal disease at the time of study and the percentage of that number out of the total number of non-diabetics involved in the study. 2) To compare values between groups, the proportion of diabetics with periodontal disease against the proportion of non-diabetics with periodontal disease was assessed. Also, the proportion of diabetics with severe periodontitis against the proportion of non-diabetics with severe periodontitis was assessed. 3) Chi-squared was used to test the association between groups with the null hypothesis that “Diabetes was not statistically significantly associated with periodontal disease.” 4) To measure the prevalence of oral hygiene practices amongst diabetics we found the number of diabetic patients in this study who had various levels of the oral hygiene practices mentioned (materials used to clean teeth, frequency of cleaning teeth, materials used to clean the interdental space, frequency of cleaning the interdental space, last visit to the dentist), and afterward calculated the percentage of each of the levels of those variables for the oral hygiene practices. To measure the prevalence of oral hygiene practices amongst non-diabetics, we found the number of non-diabetic patients in this study who had various levels of the oral hygiene practices mentioned (materials used to clean teeth, frequency of cleaning teeth, materials used to clean the interdental space, frequency of cleaning the interdental space, last visit to the dentist), and afterward calculated the percentage of each of the levels of those variables for the oral hygiene practices. The oral hygiene practices between the diabetics and non-diabetics were compared using Chi-squared as a test statistic to test the null hypothesis; “Oral hygiene practices amongst diabetics and non-diabetics are not different.” 5) Among the diabetic patients, we also utilized Chi-squared to test: a) if the duration of diabetes diagnosis was significantly associated with the severity of periodontal disease, and b) if their blood glucose control was significantly associated with the severity of periodontal disease. P-values < 0.05 were considered to be statistically significant at a 95% confidence interval. Logistic regression analysis was utilized to determine the direction of association variables.

**Ethical approval:** ethical clearance for the study protocol was obtained from the health research ethics committee of a large tertiary hospital in Lagos with the number ADM/DCST/HREC/APP/215. Verbal and written informed consent were obtained from each participant before their participation in the study which was voluntary.

## Results

**Socio-demographic characteristics:** there was a higher proportion of female diabetics 72 (65.5%) compared to male diabetics 38 (34.5%), and also female non-diabetics 62 (56.4%) compared to male non-diabetics 48 (43.65). The highest frequency of diabetic respondents fell within the age group of 60 to 69 years 41 (37.3%), while the highest frequency of the non-diabetics respondent 41(37.3%) fell within the age group of 40 to 49 years and was statistically significant <0.001. The mean ages of the respondents were 60.70 ± 10 for the diabetics and 54.45 ± 10 for the non-diabetics (Table 1).

**Table 1:** socio-demographic characteristics of respondents

	Periodontal status				
Variables	Diabetics, n=110; frequency (%)	Non-diabetics, n=110; frequency (%)	x^2^	df	P-value
**Sex**					
Female	72(65.5)	62(56.4)	1.909	1	0.214
Male	38(34.5)	48(43.6)			
**Age group (years)**					
40-49	17(15.5)	41(37.3)	17.366	4	0.001*
50-59	31(28.2)	33(30.0)			
60-69	41(37.3)	26(23.6)			
70-79	18(16.4)	9(8.2)			
80-89	3(2.7)	1(0.9)			
Mean ± SD	60.7±10	54.4±10			
**Religion**					
Christianity	96(87.3)	85(77.3)	5.866	2	0.051
Islam	13(11.8)	18(16.4)			
Traditional	1(0.9)	7(6.4)			
**Ethnicity**					
Hausa	1(0.9)	8(7.3)	5.837	3	0.124
Igbo	45(40.9)	40(36.4)			
Others	8(7.3)	7(6.4)			
Yoruba	56(50.9)	55(50.0)			
**Marital status**					
Divorced	1(0.9)	3(2.7)	5.101	4	0.244
Married	86(72.8)	91(82.7)			
Single	5(4.5)	6(5.5)			
Widowed	18(16.4)	9(8.2)			
Others	0(0.0)	1(0.90)			

**Oral hygiene practices:** majority of the diabetic patients 90 (81.8%) and non-diabetic respondents 92 (83.6%) used toothbrush and paste in cleaning their teeth. Nearly three quarters of diabetic group brushed once daily 80 (72.7%) compared to about half 59 (53.6%) of the non-diabetes group, and majority used toothpick in cleaning the space between their teeth 74 (67.3%) among diabetes group while 73 (66.4%) among non-diabetes group did same (Table 2).

**Table 2:** oral hygiene habits among diabetic and non-diabetic respondents

	Periodontal status		x^2^	df	P-value
Variables	Diabetes (%), n=110	Non-diabetes (%), n=110			
**^+^Teeth cleaning materials**					
Chewing stick	3(2.7)	6(5.5)	1.843	2	0.426
Toothbrush and paste	90(81.8)	92(83.6)			
Chewing stick and toothbrush	17(15.5)	12(10.9)			
**Frequency of teeth cleaning**					
Once daily	80(72.7)	59(53.6)	12.645	3	0.002*
Twice daily	26(23.6)	50(45.5)			
More than twice daily	3(2.7)	1(0.9)			
Others	1(0.9)	0(0.0)			
Not available	14(14.6)	8(8.3)			
**^+^Materials used to clean the interdental space**					
Dental floss	15(13.6)	15(13.6)	3.952	4	0.416
Toothpick	74(67.3)	73(66.4)			
Interdental brush	1(0.9)	5(4.)			
Broom stick	7(6.4)	9(8.2)			
Nothing	13(11.8)	8(7.3)			
**Frequency of cleaning the interdental space**					
Once daily	7(7.3)	8(7.8)	7.661	4	0.154
More than once daily	8(8.3)	10(9.8)			
Every time I eat	16(15.6)	24(23.5)			
When food gets in	66(68.8)	58(56.9)			
Others	0(0.0)	2(2.0)			
I don’t clean	14(12.7)	8(7.3)			
**Last visit to the dentist (months)**					
Less than 6 months ago	14(12.7)	15(13.6)	5.352	5	0.376
6-12 months	5(4.5)	5(4.5)			
12-18 months	4(3.6)	5(4.5)			
18-24 months	10(9.1)	4(3.6)			
More than 24 months	29(26.4)	21(19.1)			
Never	48(43.6)	60(54.5)			

* Statistically significant, X^2^-Chi-squared, ^+^multiple answers allowed

**Prevalence and severity of periodontitis:** prevalence of periodontitis was very high in both groups, however the percentage of diabetics was much higher (diabetic 90.9%, non-diabetic 71.8%). This finding was statistically significant (p=0.000) (Table 3). Less than half (41.8%) had gingivitis/mild periodontal disease (codes 1 and 2) from the diabetic group while similarly over a third (40.0%) had gingivitis in the non-diabetic group. Almost half 54 (49.1%) had severe periodontitis (codes 3 and 4) from the diabetic group as compared to nearly a third 35 (31.8%) who had severe periodontitis from among the non-diabetic group. Only 10 (9.1%) among the diabetics had healthy gums compared to 31 (28.2%) of the respondents in the non-diabetics who had healthy gums. The severity of periodontitis between diabetic and non-diabetic is statistically significant (p=0.001) (Table 4).

**Table 3:** prevalence of periodontal diseases among diabetic and non-diabetic respondents

	Periodontal status		x^2^	df	P-value
Variables	Diabetes (%), N=110	Non-diabetes (%), N=110			
Presence of periodontal disease	100(90.9)	79(71.8)	11.251	1	0.000*
Absence of periodontal disease	10(9.1)	31(28.2)			

* Statistically significant, X^2^-Chi-squared

**Table 4:** comparison of the severity of periodontitis among diabetic and non-diabetic respondents

	Periodontal status		x^2^	df	P-value
	Diabetes (%), n=110	Non-diabetes (%), n=110			
Periodontal status					
Healthy	10(9.1)	31(28.2)	14.857	2	0.001*
Mild periodontitis (gingivitis)	46(41.8)	44(40.0)			
Severe periodontitis	54(49.1)	35(31.8)			

* Statistically significant, X^2^-Chi-squared

**Association between years with diabetes, HbA1c and severity of periodontitis:** diabetic patients who had mild (code 1 and 2) periodontitis 22 (45.8%) and severe (code 3 and 4) periodontitis 22 (45.8%) exhibited good glycaemic control, whereas those with poor glycaemic control presented with mild periodontitis 18 (36.0%) and severe periodontitis 27 (54.0%). Most of the diabetic patients had between 1 to 10 years in duration with majority having severe periodontitis 35 (46.1%) and only a few of them presenting with healthy gums (Table 5).

**Table 5:** association between years with diabetes, HbA1c and severity of periodontitis

	Diabetes, N= 110		
Variable	Periodontal status (%)		
	Healthy	Mild	Severe
**Years with diabetes**			
1-10	8(10.5)	33(43.4)	35(46.1)
11-20	2(9.5)	9(42.9)	10(47.6)
21-30	0(0.0)	3(30.0)	7(70.0)
31-40	0(0.0)	0(0.0)	2(100.0)
41-50	0(0.0)	1(100.0)	0(0.0)
**TOTAL**	10	46	54
	X^2^ = 5.784	df = 2	p = 0.745
**Hemoglobin A1C (HbA1C**)			
Good	4(8.3)	22(45.8)	22(45.8)
Poor	5(10.0)	18(36.0)	27(54.0)
N/A	1(8.3)	6(50.0)	5(41.7)
**TOTAL**	10	46	54
	X^2^ = 1.544	df = 4	p = 0.854

**Table 6:** odds ratio for presence and severity of periodontitis with diabetics at 95% confidence intervals

Variable	Odds ratio	95% (CI)	P-value
**Presence of periodontal disease**			
Present	3.580	1.647 - 7.780	0.001*
Absent	REF(1.0)		
**Severity of periodontal disease/periodontal status**			
Healthy	REF(1.0)		
Mild periodontitis (gingivitis)	3.241	1.422 - 7.388	0.005*
Severe periodontitis	4.783	2.085 - 10.969	0.000*

* Statistically significant

## Discussion

Our study revealed that there was a higher prevalence rate of mild/severe periodontitis among diabetics (90.9%) compared to non-diabetics (71.8%), which was statistically significant p<0.000. This is consistent with a study conducted among Mauritians with a prevalence of (100%) periodontitis in diabetes compared with non-diabetics in which 98% suffered from gingivitis (mild periodontal disease) but inconsistent with studies in Tamil Nadu in which the prevalence of chronic periodontitis among the diabetic group was 45.9%, while it was 37.8% among the non-diabetics [23]. Furthermore, a study conducted among diabetic and non-diabetic patients in Korea by Mihee Hong *et al*. recorded the prevalence of periodontitis in the diabetic group as 43.7%, and 25% in the non-diabetics group [15], which is of a lower prevalence and can be possibly attributed to their better knowledge and health-seeking behavior considering Korea being a developed country [15].

Furthermore, our study revealed that 54 (49.1%) of diabetics compared to 35 (31.8%) of non-diabetics presented with severe periodontitis which is statistically significant (p=0.001). This is in agreement with Matu N *et al*. [24] in South Africa and also in line with a study conducted in Tamil Nadu with the severity of periodontitis higher among 38.8% of diabetics as compared to 29.9% of non-diabetics [23] but in contrast to that recorded in a study done at Ile-Ife, Nigeria, which recorded the prevalence of severe periodontitis (CPITN code 4) in the diabetic as 20% compared to non-diabetic patients 15% with no statistically significant difference [8]. The prevalence from previous studies is lower compared to our study possibly due to better oral hygiene awareness and frequent dental visits. Also in contrast to the study done in India 60% of diabetic patients compared to 16% of non-diabetic patients examined were found to have periodontitis [25]. This result can be attributed to the few numbers of sample size involved in that study [23].

When the glycaemic control was evaluated, 45.8% of diabetic patients with severe periodontitis had good HbA1c control whereas 8.3% who had healthy gingiva had good HbA1c. There is no statistically significant association between HbA1c control and the severity of periodontitis which is consistent with a study carried out by Tervonen and Karjalainen who reported that the level of periodontal health in diabetic patients with good or moderate control of their condition was similar to that in the non-diabetic controls. Those with poor control had more attachment loss and were more likely to exhibit recurrent disease [9]. Additionally, reports from this study show that an increase in duration is directly related to the severity of periodontitis though this was not statistically significant. The vast majority of diabetic patients with a duration of 1-10 years had severe periodontitis (46.1%) while none of the patients with a duration of 41-50 years had severe periodontitis, though a study done in Saudi Arabia reported a statistically significant relationship between duration or diagnosis of diabetes and the severity of periodontal inflammation [26,27]. According to Al-Shammari *et al*. [28], periodontal parameters (mobile tooth and clinical attachment loss) were significantly higher in patients with a longer duration of type 2 diabetes (≥ 5 years) compared with individuals with a shorter duration of diabetes (< 5 years), the duration of prediabetes and type 2 diabetes in group 1 and group 2 patients was approximately 2 years and 3 years respectively.

**Study strengths, limitations, and areas for future research:** this study suggests that periodontitis has a direct relationship with glycaemic control. In addition, within the limitations of this study, preference was not made for patients with a longer duration of diabetes. Further studies should be carried out to ascertain when diabetic patients are out of control and their relationship with the severity of periodontitis. Furthermore, biomarkers such as interleukins, matrix metalloproteinases (MMP), cytokines, and collagenases which are implicated in periodontitis will also be considered.

## Conclusion

This study suggests that diabetes is a significant risk factor for periodontitis, and the risk of periodontitis is greater if glycaemic control is poor; there is an increased risk of periodontitis and alveolar bone loss with poorly controlled diabetics who are almost at risk for the other macrovascular and microvascular complications There is a need for targeted education regarding oral hygiene to reduce this preventable condition. Management of diabetes leading to good glycaemic control would be an important strategy for the prevention of or progression of periodontitis to prevent further bacterial invasion which can further increase the risk. Long-term dental follow-up visits are required for all those with diabetes.

### What is known about this topic

- *There is emerging evidence to support the existence of a two-way relationship between diabetes and periodontitis;*
- *There is an increased prevalence of periodontitis in diabetic patients*.

### What this study adds

- *This study provides data that establish an updated estimate of those diabetic and non-diabetic patients affected by periodontitis; there is mild to severe periodontitis in patients with diabetes within the duration of 1 to 10 years;*
- *There is compromised periodontal status in patients with good glycated haemoglobin;*
- *The study established a link between periodontitis and diabetes and it reveals the high prevalence of periodontitis in diabetic patients with the sense of integrating periodontal treatment in patients with diabetes*.
